# Non-operative management of blunt splenic injury: is it really so extensively feasible? a critical appraisal of a single-center experience

**DOI:** 10.11604/pamj.2019.32.52.15022

**Published:** 2019-01-30

**Authors:** Pietro Fransvea, Gianluca Costa, Giulia Massa, Barbara Frezza, Paolo Mercantini, Genoveffa BaIducci

**Affiliations:** 1 Faculty of Medicine and Psychology, University of Rome "La Sapienza" St Andrea Hospital, Italy

**Keywords:** Spleen, blunt trauma, selective non operative management

## Abstract

**Introduction:**

The spleen is one of the most commonly injured organ following blunt abdominal trauma. Splenic injuries may occur in isolation or in association with other intra-and extra-abdominal injury. Nonoperative management of blunt injury to the spleen has become routine in children. In adult most minor splenic injuries are readily treated nonoperatively but controversy exists regarding the role of nonoperative management for higher grade injuries above all in multi-trauma patients. The aim of this study is the assessment of splenic trauma treatment, with particular attention to conservative treatment, its limits, its efficiency, and its safety in multi-trauma patient or in a severe trauma patient.

**Methods:**

The present research focused on a retrospective review of patients with splenic injury. The research was performed by analyzing data of the trauma registry of St. Andrea University Hospital in Rome. The St. Andrea University Hospital trauma registry includes 1859. The variables taken into account were spleen injury and general injuries, age, sex, cause and dynamic of trauma, hemoglobin, hematocrit, white blood cells count, INR, number and time blood transfusion, hemodynamic stability, type of treatment provided, hospitalization period, morbidity and mortality. Assessment of splenic injuries was evaluated according to Abbreviated Injury Scale (AIS).

**Results:**

The analysis among the general population of spleen trauma patients identified 68 patients with a splenic injury representing the 41.2% of all abdomen injury. The Average age was of 37.01 ± 17.18 years. The Average ISS value was of 22.88 ± 12.85; mediana of 24.50 (range 4-66). The average Spleen AIS value was of 3.13 ± 0.88; mediana 3.00 (range 2-5). The overall mortality ratio was of 19.1% (13 patients). The average ISS value in patients who died was of 41.92 ± 12.48, whereas in patients who survided was of 23.33 ± 10.15. The difference was considered to be statistically significant (p <0.001). The relashionship between the ISS and AIS values in patients who died was considered directly proportional but not statistically significant (Pearson test AIS/ISS = 0.132, p = n.s.). The initial management was a conservative treatment in 27 patients (39.7%) of them 4 patients (15%) failed, in the other 41 cases urgent splenectomies were performed. The average spleen AIS in all the patients who underwent splenectomy was 3.61 ± 0.63 whereas in the patients who were not treated surgically was 2.42 ± 0.69. The difference was deemed statistically significant (p <0.001).

**Conclusion:**

Splenic injury, as reported in our statistic as well as in literature, is the most common injury in closed abdominal trauma. Nonoperative management of blunt injury to the spleen in adults has been applied with increasing frequency. However, the criteria for nonoperative management are controversial. The preference of a conservative treatment must be based on the hemodynamic stability indices as well as on the spleen lesion severity and on the general trauma severity. The conservative treatment represent a feasible and safe therapeutic alternative even in case of severe lesions in politrauma patients, but the choice of the treatment form requires an assessment for each singular case.

## Introduction

The treatment of blunt splenic injuries (BSI) has changed significantly during the last 30 years with the non-operative management (NOM) that has become a standard of care both in children and in adults [1]. However, a number of issues regarding the management of adult patients with BSI are still unresolved [2]. Presently the criteria for NOM of BSI included hemodynamic stability on admission or after initial resuscitation, no peritoneal signs or any associated injuries necessitating laparotomy. The presence of multiple injuries, high-grade splenic injury, a large haemoperitoneum, age and high Injury Severity Score (ISS) are reported as risk factors for failure of NOM [3]. The evaluation of severe trauma patients, the so called politrauma, is paramount because up to 30% of such patients have abdominal injuries. High rate of concomitant injuries occur in patients with blunt splenic trauma, reflecting the epidemiology of trauma injuries due to road traffic accidents, the most common mechanism of injury in western countries. The feasibility, indications and risks of selection for NOM in such instances are less clear. Moreover the clinical decision to perform NOM is reportedly influenced by others various factors such as surgeon training and experience, available non-physician stuff and resources and hospital type. The aim of this study is to evaluate our current practices regarding BSI in order to assess meanly the real rate of feasibility and safety of NOM in multi-trauma patient or in a severe trauma patient.

## Methods

A retrospective trauma registry review was performed by analysing data from the University Hospital Sant'Andrea in Rome. This hospital is a tertiary and teaching hospital located in a large urban area accounting for 400.000 people. The Trauma registry Study Project provided for the enrolment of patients over 16 years of age, victims of either blunt or penetrating trauma and burn injury has been extensively previously described [4]. The clinical records of all blunt abdominal trauma observed between January 2012 and December 2016 were analysed. Assessment of splenic injuries was evaluated according to Abbreviated Injury Scale (AIS), using the 2005 version of the AIS-CD, updated in 2008, Association for the Advancement of Automotive Medicine-AAAM), (Barrington, IL, USA) codes (544299.2 to 544240.3). Variables considered for analysis were: age, gender, cause and mechanism of injury, Systolic Blood Pressure (SBP), Glasgow Coma Scale (GCS), White Blood Cell Count (WBC), haemoglobin, haematocrit, INR, number and time blood transfusion and clinical abdominal signs at the admission, hemodynamic stability, type of associated lesions, Abbreviated Injury Scale (AIS) for abdominal, head, chest and extremities. Injury Severity Score (ISS) was calculated to evaluate the entire body trauma severity. CT scan findings at admission, time from Emergency Room (ER) arrival to Operating Room (OR), total elapsed time, type of treatment provided (NOM versus others), length of hospital stay (LOS). Morbidity and mortality were also retrieved from the database. Hemodynamic stability was based on Systolic Blood Pressure (SBP), on Diastolic Blood Pressure (DBP), and on the Shock Index (SI) at time zero, at three-hour intervals during the first 12 hours, at 18 hours, 24 hours and 36 hours. Patients with SI values not more than 0,8099 and/or with systolic blood pressure more than 90mHg were considered hemodynamically stable. Total elapsed time was defined as the time from the onset of trauma to the time of diagnosis regardless of the treatment. The comparison of different kinds of treatment was performed by analyzing the percentage of non-operative management (NOM) and operative with regard to the evaluation of success and failure rates. We considered as a NOM any treatment initially adopting after three hours of Emergency Department admission the preservation of the spleen regardless to the performance of any other invasive procedure, such as embolization. Morbidity was evaluated according to Clavien-Dindo (C-D) Classification [5, 6].

Mortality was identified as any death occurring within the first 30 days from trauma or during the entire hospital stay if it could be logically linked to the event. In order to evaluated the appropriateness, the efficacy and the safety of the treatment provided each patients' clinical course were submitted to a clinical audit. Since the two main senior authors (GC, GB) could have been involved in the management of a number of clinical cases included in the study, they were blinded for patient's name, identity number and his or her clinical course. The investigators were also blinded as regards the name of the surgeons and physicians involved in the diagnosis and treatment. On the basis of blind setting a different senior emergency and trauma surgeon was asked to determine if patient could be suitable for NOM. A grade by grade comparison between the treatments was then carried out. If any data were missing, either a follow-up was conducted by phone, or information was requested from sources in the region such as hospitals patients were transferred to, general practitioners, registry office and the police. The research was undertaken according with the Italian Privacy Laws concerning collection, storage and analysis of private data for scientific purpose. Approval from the University Hospital Sant'Andrea Institutional Research Ethics Boards was not required because of the retrospective and anonymous study design. Statistical analysis was carried out using the 17.0 version of the PASW Statistics Programme (SPSS Italy, Bologna) for MacOsX. Data were encoded either as continuous descriptive data or as categorical dichotomous covariate (yes/no). Descriptive data are summarized as mean ± SD, median [range/interquartile), or percentage (%). The separate effect of each variable upon outcome was examined individually in a univariate analysis considering morbidity and mortality as dependent variable. The one-way analysis of variance (ANOVA) test, the chi-square test, the Spearman's test and the t Student's test or Mann-Whitney statistical test were used when appropriate. All variables with p value < 0.20 in the univariate analysis were included in a multivariate logistic binary regression analysis by forward stepwise and backward stepwise method. CT overall diagnostic accuracy was calculated with on-line calculators. P < 0.05(two-tailed) was considered statistically significant. The 95% confidence interval (CI) and the odds ratio (OR) were reported when appropriate.

## Results

There were 3519 patients with chief complaints of trauma or burns. A total of 1859 cases that satisfied the inclusion criteria, were entered in the database for the study, and all patients with abdominal trauma were further selected. The Average age of 1859 patients, included in the trauma registry data of St. Andrea University Hospital in Rome, was of 61.05 ± 73.66 years; 1028 (55.29%) were male and 822 (44.2%) were female. According to the color code triage, we observed 351 red color codes (18.8%), 1441 yellow color codes (7.51%) and 64 green color codes. The color code triage has not been used for two patients, since they had been transferred from other health facility. Patients were mostly victims of road traumas (845 cases, 45.5%) followed by victims of domestic traumas (789 patients, 44.4%). Sport injuries and work-place injuries occurred less frequently (Table 1). 31 patients were victims of penetrating injuries (firearms wounds and cold steel wounds) and 10 (0.53%) were burn patients. In 1501 cases the most commonly encountered lesion were: orthopedic injuries (80,74%) followed by thorax lesions in 391 patients (21.03%) and by neurosurgical lesions in 274 patients (14.73%). Abdomen traumas were recorded in 165 patients (8.87%). Incidence of different injuries is reported in Table 1. The Average ISS value was 10.57 ± 8.03. The average mortality rate and the average morbidity rate were of 3.81% (71 patients) and of 8.2% (154 patients) respectively. The analysis among the general population of abdominal trauma patients identified 68 patients with a splenic injury representing the 41.2% of all abdomen injury. The average age was of 37.01 ± 17.18 years. There were more men than women (56 cases, 82.35% and 12 cases, 17.64% respectively) In 63 cases (92.64%) patients were mostly road trauma victims, followed by 4 sport accidents (5.88%) and by 1 domestic trauma (1.47%). Accurate records of seat belt usage and airbag deployment were not available in sufficient cases to report in a meaningful way on these items. The average ISS value was of 22.88 ± 12.85; mediana of 24.50 (range 4-66). The most common associated lesions are shown in Table 1.

**Table 1:** Mechanism of injury and type of injuries

Mechanims of injury: 1859 pt.	%	Type of injury (%)	General trauma population (1859)	Spleen trauma population (68)
				
Road traumas	45.4	Orthopedics	80.7	61.8
				
Domestic traumas	42.4	Thoracic	21.0	73.5
				
Sport injures	4.6	Neurosurg.	14.7	23.5
				
Work-place injuries	2.9	Max-fac	11.4	11.4
				
Others	2.0	Abdominal	8.9	69.1
				
		Plastic Surg.	4.9	5.9
				
		Vascular	2.4	5.9

The average Spleen AIS value was of 3.13 ± 0.88; mediana 3.00 (range 2-5). The distribution of patients with respect to the Splenic AIS grading showed an AIS = 2 in 20 cases (29.4%); an AIS = 3 in 21 cases (30.9%); an AIS = 4 in 25 cases (36.8%) and an AIS = 5 in 2 cases (2.9%). The overall mortality rate was of 19.1% (13 patients). The average ISS value in patients who died was of 41.92 ± 12.48, whereas in patients who survived was of 23.33 ± 10.15. The difference was considered to be statistically significant (p < 0.001). The difference between the average spleen AIS value in patients who died (3.31 ± 0.85) and the average spleen AIS value observed in patients who survived was not considered statistically significant. The relationship between the ISS and AIS values in patients who died was considered directly proportional but not statistically significant (Pearson test AIS/ISS=0.132, p = n.s.). The morbidity rate was 36.8% (25 patients) and the more frequent complications are shown in Table 2. The average ISS in patients with complications and those with a regular disease course was 29.48 ± 10.42 and 25.33 ± 14.13 respectively. The difference between the two values was not considered statistically significant. The ISS value and the Age value stratified according to the AIS severity classes are shown in Table 3. The differences were considered statistically significant only as regard to ISS (p < 0.03). The initial management was a conservative treatment in 27 patients (39.7%), with abdominal CT scan as part of their secondary survey at admission, one of them underwent embolization of splenic artery (3.7%), an another of them underwent video laparoscopic hemostasis (3.7%). In the other 41 with a positive FAST evaluation cases urgent splenectomies were performed due to hemodynamic instability, whereas in 4 cases it was not possible to use any kind of treatment because of the severe patients' conditions or of the sudden patient's death. In the group of patients treated with a conservative management, 5 (18.5%) delated splenectomy occurred. The average time elapsed between admission to the emergency department and sudden splenectomy was of t = 114 ± 61.8 minutes; mediana 100 (range 30-240). The 5 patients in whom the conservative treatment failed underwent surgery within 8, 19, 36 hours or 6 and 7 days from the trauma.

**Table 2:** Type of complications

Type of Complications	n. cases (%)
Pulmonary	21 (30.8)
Infective	6 (8.8)
Acute renal failure	5 (7.3)
Hemorragic shock	3 (4.4)
Intestinal occlusion	3 (4.4)
Others	17 (25)

**Table 3:** ISS value and the age value stratified according to the AIS severity classes

	N	Avarange	Standard dev.	Standard Err.	Conf.Interv Upper Limit	Conf.Interv Lower Limit	Min	Max
***age***								
2	20	39.3	19.315	4.319	30.26	48.34	17	76
3	21	36.62	17.814	3.887	28.51	44.73	16	68
4	25	37.68	16.530	3.306	30.86	44.50	16	85
5	2	39.00	5.657	4.000	11.82	89.82	35	43
ToT	68	37.87	17.321	2.101	33.68	42.06	16	85
***I.S.S***								
2	20	20.65	12.197	2.727	14.94	26.36	4	50
3	21	30.67	13.086	2.856	24.71	36.62	10	50
4	25	27.56	11.794	2.359	22.69	32.43	16	66
5	2	41.00	.000	.000	41.00	41.00	41	41
ToT	68	26.88	12.858	1.559	23.77	29.99	4	66

The average spleen AIS in all the patients who underwent splenectomy was 3.61 ± 0.63 whereas in the patients who were not treated surgically was 2.42 ± 0.69. The difference was deemed statistically significant (p < 0.001). The AIS value was of 3 in four cases and of 2 in one case. The average ISS in all the patients who underwent splenectomy was 30.88 ± 11.84 whereas in the non-operatively treated patients was of 20.81 ± 12.12. The difference between ISS median values was deemed statistically significant (p < 0.001). The comparison between average ISS, average spleen AIS, PA,FC, Hb and SI values reported at time zero and within 3 hours in patients who underwent splenectomy and in patients managed non operatively (or conservatively treated patients), is shown in Table 4. The differences were statistically significant as regard to PA at time zero (p = 0.029), PA within 3 hours (p = 0.02), Hb at time zero (p < 0.02), Hb within 3 hours (p < 0.01), and SI at time zero (p = 0.01). From the "blind" analysis, 36 (52.9%) patients could be scheduled for NOM, but only in 27 (39.7%) of cases NOM was effectively performed. Of the 36 non-operative candidates, stratified in accordance with spleen AIS, 14 cases (38.8%) had a grade 2 injury, 8 cases (22.2) had a grade 3 injury, 13 cases (36.1%) had a grade 4 injury and 1 case (2.7%) had a grade 5 injury. The same stratification was performed in the 27 patients who were non-operatively treated. The differences are shown in Table 5 and in Table 6. The comparison of spleen AIS, ISS, PA,FC, Hb and SI average values reported in time zero and after 3 hours, in the 36 patients who were candidates for non-operative management and who were divided in non-operative treated patients and in patients who underwent splenectomy is shown in Table 7. The differences were deemed statistically significant only for spleen AIS (p < 0.01) and ISS (p < 0.06), but the patients who underwent splenectomy had lower Hb values and systolic blood pressure either at time zero than after 3 hours even if differences were not deemed statistically significant. The multivariate analysis does not demonstrate independent variables statistically associated with the failure of conservative treatment.

**Table 4:** Comparison between patients who underwent splenectomy and patients managed non-operatively

	N.	Avarange	Standard dev	Standard Err	Conf.Inter 95% Lower Limit	Conf.Interv 95% Upper Limit	Min	Max
***AIS***								
0	41	3.82	0.628	0.098	3.41	3.81	2	5
1	27	2.4	0.694	0.134	2.13	2.68	2	4
ToT	68	3.03	0.879	0.107	2.92	3.35	2	5
***ISS***								
0	41	26.40	11.839	1.849	27.14	34.61	10	66
1	27	17.14	12.124	2.333	16.02	25.61	4	50
ToT	68	21.00	12.858	1.559	23.77	29.99	4	66
***Hb0***								
0	41	12.533	2.4772	0.3869	11.169	12.733	7.2	16,6
1	27	13.162	1.8440	0.3549	12.648	14.107	8.5	16,9
ToT	68	12.900	2.3404	0.2838	11.951	13.084	7.2	16,9
***Hb3***								
0	37	11.137	2.1965	0.3611	9.776	11.240	5.2	14.5
1	27	12.276	2.0352	0.3917	11.199	12.809	6.6	14.2
ToT	64	11.824	2.2407	0.2801	10.579	11.699	5.2	14.5
***Hr0***								
*0*	41	78.87	21.453	3.350	87.64	101.19	40	130
1	27	85.10	12.016	2.313	82.88	92.38	65	120
ToT	68	82.50	18.493	2.243	87.24	96.20	40	130
***Hr3***								
0	28	87.75	14.534	2.747	82.11	93.39	65	119
1	20	88.90	18.904	4.227	80.05	97.75	65	130
ToT	48	88.23	16.314	2.355	83.49	92.97	65	130
***SBP0***								
0	41	102.80	26.672	4.166	94.39	111.22	40	175
1	27	122.44	19.041	3.664	114.91	129.98	80	155
ToT	68	110.60	25.674	3.113	104.39	116.82	40	175
***SBP3***								
0	27	104.59	19.346	3.723	96.94	112.25	70	160
1	21	117.19	16.333	3.564	109.76	124.63	80	140
ToT	48	110.10	18.985	2.740	104.59	115.62	70	160
***Shock Index***								
0	41	1.0267	0.55098	0.08605	0.8528	1.2006	0.38	3.25
1	27	0.7359	0.17462	0.03361	0.6668	0.8050	0.50	1.22
ToT	68	0.9112	0.46219	0.05605	0.7994	1.0231	0.38	3.25

**Table 5:** Possibility and type of treatment

AIS count (%)	Poss. NOM	Poss NOM	
	0	1	ToT
2	6 (30.0%)	14	20
3	13 (61.9%)	8 (38.1%)	21 (100%)
4	12 (48.0%)	13 (52.0%)	25 (100%)
5	1(50.0%)	1(50.0%)	2 (100%)
ToT	32 (47.1%)	36 (52.9%)	68 (100%)
**AIS Count (%)**	**NOM**	**NOM**	
	0	1	ToT
2	1 (5.0%)	19 (95.0%)	20 (100%)
3	16 (76.2%)	5 (23.8%)	21 (100%)
4	22 (88.0%)	3 (12.0%)	25 (100%)
5	2 (100%)	0 (0%)	2 (100%)
ToT	41 (60.3%)	27 (39.7%)	68 (100%)

**Table 6:** Haemodinamical status and type of treatment

	Hemodynamically stable NOM	Hemodynamically unstable NOM	Hemodynamically stable Surgery
***AIS***			
2	14	5	0
3	4	1	4
4	3	0	10
5	0	0	1
ToT	21	6	15

**Table 7:** Variables analyzed in patients with splenic trauma

	N.	Avarange	Standard dev	Standard Err	Conf.Inter 95% Lower Limit	Conf.Interv 95% Upper Limit	Min	Max
***AIS***								
0	15	3.80	0.561	0.145	3.49	4.11	3	5
1	21	2.48	0.750	0.164	2.13	2.82	2	4
ToT	36	3.03	0.941	0.157	2.71	3.35	2	5
***ISS***								
0	15	26.40	10.602	2.737	20.53	32.27	16	50
1	21	17.14	8.033	1.753	13.49	20.80	4	38
ToT	36	21.00	10.162	1.694	17.56	24.44	4	50
***Hb0***								
0	15	12.533	1.9389	0.5006	11.460	13.607	9.0	16.6
1	21	13.162	1.6842	0.3675	12.395	13.929	8.5	16.1
ToT	36	12.900	1.7954	0.2992	12.293	13.507	8.5	16.6
***Hb3***								
0	14	11.136	1.8604	0.4972	10.062	12.210	7.6	14.5
1	21	12.276	1.5703	0.3427	11.561	12.991	8.3	14.0
ToT	35	11.820	1.7593	0.2974	11.216	12.424	7.6	14.5
***Hr0***								
*0*	15	78.87	9.782	2.526	73.45	84.28	64	100
1	21	85.10	10.099	2.204	80.50	89.69	65	110
ToT	36	82.50	10.308	1.718	79.01	85.99	64	110
***Hr3***								
0	9	86.44	15.412	5.137	74.60	98.29	70	110
1	16	85.31	17.192	4.298	76.15	94.47	65	130
ToT	25	85.72	16.255	3.251	79.01	92.43	65	130
***SBP0***								
0	15	125.73	21.036	5.431	114.08	137.38	100	175
1	21	130.05	12.929	2.821	124.16	135.93	108	155
ToT	36	128.25	16.648	2.775	122.62	133.88	100	175
***SBP3***								
0	10	112.90	26.164	8.274	94.18	131.62	70	160
1	17	119.76	13.170	3.194	112.99	126.54	90	140
ToT	27	117.22	18.844	3.627	109.77	124.68	70	160
***Shock Index***								
0	15	0.646910	1430271	0.0369294	0.567704	0.726115	0.3771	0.8000
1	21	0.657309	0.0752494	0.0164208	0.623056	0.691562	0.5000	0.7857
ToT	36	0.652976	0.1069833	0.0178306	0.616778	0.689174	0.3771	0.8000

## Discussion

Abdominal organ injuries are a frequent cause of morbidity and mortality in trauma patients, with the abdomen involved in 7-20% of cases [7-9]. In western countries, the most frequent cause of abdominal trauma is traffic collisions with over half of the injured patients being car drivers, motorbike drivers and pedestrians. Most of involved people are young, with mean age being 30-40 years and a predominance of male gender [10-16]. Spleen injuries, as reported from our series and from literature, represent the most common event among blunt abdominal traumas [17-21], and can be caused by a contusive mechanism directly on the abdominal wall or to the base of the left hemithorax, but also by recoil due to a sudden acceleration or deceleration with dispersion of high energies. Spleen trauma prognosis is strictly related to the severity of the splenic injury and of the trauma in general. A correct evaluation of the trauma severity helps the patient be triaged properly and can therefore influence the clinical course and management. Interestingly, in our research in almost half of the cases of severe trauma, an abdominal injury was seen too. Also, abdominal traumas were frequently associated to bone fractures and thorax and central nervous system injuries. This is expected as almost all patients undergoing severe traumas and with an abdominal injury, are victims of traffic accidents and therefore, due to the major dynamic of the event, present more injuries at the same time. Our data are in line with those previously reported in the literature in terms of age, sex, causes and severity of the trauma in case of splenic injury, which is almost always associated to a severe trauma or a polytrauma, in patients aged 35-45, mostly males [17, 18].

The analysis of the injuries associated to spleen ones showed how the most frequent are those regarding the thorax and bones and those concerning other abdominal organs, followed by injuries concerning the neurosurgical, maxillofacial, plastic and vascular field. These data are new and therefore not comparable to previous literature; in fact, the latter lacks on information about the type of injuries associated to spleen trauma and, therefore, it is hard to evaluate an impact in terms of morbidity and mortality. The conservative management of spleen trauma started spreading in the 60s, initially with spleen-preserving surgeries such as partial resections or hemostatic splenorraphies. In the following years, Para surgical treatments took the lead, with interventional radiologists performing splenic artery embolization with the aim of reducing the bleeding and avoiding non-therapeutic laparotomies. Nowadays, a non-operative treatment is preferred, mostly with a "wait and see" approach. In the last 40 years, the non-operative management has almost completely replaced splenectomy and is now widely accepted as the standard care for low-grade injuries. In our experience, in line with the literature, no patient with an Abbreviated Injury Scale >3 has undergone a conservative management [22-26]. In many published case-series, about 60% of patients with splenic injuries are treated conservatively; this number is higher compared to our study (about 40%), although with a very low rate of failure [27-31]. This difference can be explained with the different severity splenic injuries seen at our center, which occurred more frequently in the context of complex general traumas, as demonstrated from the statistically significant difference between splenic AIS values and medium ISS in our two groups of patients; nevertheless, these differences are reported also in the literature. Another elements influencing the treatment choice are related to the referral hospital, with its organizing and structural abilities, in order to access the operating room in the shortest time and to utilize the most modern diagnostic instruments or to offer non-operative options such as the splenic artery embolization We, therefore, evaluated the limits of a conservative management in terms of feasibility efficacy and safety [27, 32-36]. The choice of treating conservatively a patient depends mostly on hemodynamic status on arrival at the Emergency Room [1, 37-39].

Many authors analyzed different indexes related to hemodynamics, with blood pressure, heart rate and shock index; the latter has an important role especially in cases in which heart rate and blood pressure are normal, allowing to hypothesize a risk of clinical condition worsening, due to persistent bleeding [1, 40-42]. When the patient is in shock and there are associated intra- or extra-abdominal or when hypothermia, the presence of coagulopathy or severe comorbidities represent a contraindication to conservative management, then an immediate surgical intervention is required; in these cases, splenectomy is the treatment of choice in most cases. Spleen-sparing surgical approaches are less cost-effective in terms of time and blood transfusions required and more technically challenging, therefore they can be proposed when the hemodynamic status of the patient is well controlled: partial splenectomy, splenorraphia, use of biological glue or splenic encasement with absorbable mesh represent the possible therapeutic strategies especially in children. The importance of the hemodynamic status in determining the management is clear also from our results, showing how there is a statistically relevant difference among values of blood pressure, heart rate and shock index observed in patients treated conservatively compared to those treated with splenectomy. Nevertheless, the possibility to treat the patient conservatively cannot be based only onto hemodynamic status; in fact, the analysis of our case-series showed how not all patients that could undergo a conservative management based on the hemodynamic status, have been eventually treated conservatively. This underlines the importance of considering other variables such as the severity of the injury and the presence of other associated injuries. In the statistical analysis regarding the comparison of hemodinamically stable patients treated conservatively versus those undergoing splenectomy, we observed, as expected, that there was no difference in terms of Hb levels, heart rate, blood pressure and shock index. The analysis showed, on the other hand, a statistically significant difference in terms of AIS and ISS values, both increased in patients undergoing splenectomy. After stratification of the patients based on the severity of splenic lesions, our study showed that with the increase of AIS values, the number of patients undergoing conservative treatment decreased linearly, which is in line with previous literature [30, 36].

An interesting aspect emerging from our study is that in patients with bleeding from splenic rupture with AIS equal to 2, a conservative management has been adopted in an even higher rate of cases, compared to cases who could be potentially being treated conservatively based only on hemodynamic status. This demonstrates how an early hemodynamic stabilization of polytraumatized patients and an accurate evaluation of the severity of the injuries can reduce the rate of surgical intervention. One of the risks of a conservative treatment is to end up to a splenectomy anyway. In these cases, “failure” of conservative management is considered only in case of persistent bleeding or its delayed recognition. In our experience, there have been five cases of failed conservative treatment, all, except one, were injuries with AIS >2. The exiguity of "failure" cases, although, does not allow us to evaluate which factor could be associated to the necessity to switch to a delayed splenectomy. The mortality rate of splenic injuries is not easily determined; in fact, also in literature this datum lacks. Our research shows a rate of mortality among 7-18% [43-48]. Our results show a higher rate, but deserves some consideration as it seems, as previously reported by other authors, due to causes independent to splenic lesions. The association between mortality and medium AIS of the spleen, in fact, is not statistically significant. Compared to patients alive, in deceased patients spleen injuries with medium AIS are in a context of a polytrauma with very high ISS. The analysis of a possible linear association between splenic AIS and ISS, also, was not statistically significant and we can therefore hypothesize how, in case of death, the cause of death could be related to the severity of the general trauma and not the splenic trauma per se.

Conclusion

Splenic trauma represents one of the most frequent injuries in case of abdominal trauma and is often in the context of a severe trauma or polytrauma, as happening in high energy trauma. The eligibility to conservative treatment has to be based not only on the index of hemodynamic stability but also on the severity of the splenic injury and the general severity of the trauma. The conservative management represents a feasible and safe therapeutic option also in case of severe polytraumatized patients, but the choice of treatment has to be evaluated case by case.

### What is known about this topic

- Non-operative management (NOM) has become a standard of care both in children and in adults for Blunt Splenic Trauma (BSI);
- Controversy exists regarding the role of nonoperative management for higher grade injuries above all in multi-trauma patients;
- Presently the criteria for NOM of BSIincluded hemodynamic stability on admission or after initial resuscitation, no peritoneal signs or any associated injuries necessitating laparotomy.

### What this study adds

- Assessment of splenic trauma treatment, with particular attention to conservative treatment, its limits, its efficiency, and its safety in multi-trauma patient or in a severe trauma patient;
- Others various factors such as surgeon training and experience, available non-physician stuff and resources and hospital type could influence the possibility of NOM;
- The conservative management represents a feasible and safe therapeutic option also in case of severe polytraumatized patients, but the choice of treatment has to be evaluated case by case.

## Competing interests

The authors declare no competing interests.
